# Acupuncture and tuina knowledge graph with prompt learning

**DOI:** 10.3389/fdata.2024.1346958

**Published:** 2024-04-08

**Authors:** Xiaoran Li, Xiaosong Han, Siqing Wei, Yanchun Liang, Renchu Guan

**Affiliations:** ^1^Key Laboratory for Symbol Computation and Knowledge Engineering of National Education Ministry, College of Computer Science and Technology Jilin University, Changchun, China; ^2^Zhuhai Laboratory of Key Laboratory for Symbol Computation and Knowledge Engineering of Ministry of Education, College of Computer Science and Technology of Zhuhai College of Science and Technology, Zhuhai, China

**Keywords:** prompt learning, knowledge graph, Traditional Chinese Medicine, Named Entity Recognition, Entity Relationship Extract

## Abstract

**Introduction:**

Acupuncture and tuina, acknowledged as ancient and highly efficacious therapeutic modalities within the domain of Traditional Chinese Medicine (TCM), have provided pragmatic treatment pathways for numerous patients. To address the problems of ambiguity in the concept of Traditional Chinese Medicine (TCM) acupuncture and tuina treatment protocols, the lack of accurate quantitative assessment of treatment protocols, and the diversity of TCM systems, we have established a map-filling technique for modern literature to achieve personalized medical recommendations.

**Methods:**

(1) Extensive acupuncture and tuina data were collected, analyzed, and processed to establish a concise TCM domain knowledge base. (2)A template-free Chinese text NER joint training method (TemplateFC) was proposed, which enhances the EntLM model with BiLSTM and CRF layers. Appropriate rules were set for ERE. (3) A comprehensive knowledge graph comprising 10,346 entities and 40,919 relationships was constructed based on modern literature.

**Results:**

A robust TCM KG with a wide range of entities and relationships was created. The template-free joint training approach significantly improved NER accuracy, especially in Chinese text, addressing issues related to entity identification and tokenization differences. The KG provided valuable insights into acupuncture and tuina, facilitating efficient information retrieval and personalized treatment recommendations.

**Discussion:**

The integration of KGs in TCM research is essential for advancing diagnostics and interventions. Challenges in NER and ERE were effectively tackled using hybrid approaches and innovative techniques. The comprehensive TCM KG our built contributes to bridging the gap in TCM knowledge and serves as a valuable resource for specialists and non-specialists alike.

## 1 Introduction

Acupuncture and Tuina stand as foundational therapies within Traditional Chinese Medicine (TCM), boasting centuries of esteemed practice and theoretical development. Their origins trace back to ancient China, where detailed theories and practical methodologies were documented in medical classics like the Huangdi Neijing over two millennia ago (Unschuld, 2016). Since the Neolithic era, these practices have gradually become integrated into human life (Cao, 2008) and are widely employed in clinical treatments due to their simplicity and rapid effectiveness. In recent years, acupuncture and tuina have gained increased recognition in various countries, including the United States and Europe. In 2020, a research article featured in the journal Neuron, authored by a team led by Professor Qiufu Ma from Harvard Medical School, demonstrates that acupuncture, through targeted stimulation of specific acupoints on the body's surface, can activate a range of “somatic-sensory-autonomic-target-organ” reflex pathways. This activation is capable of effecting immune-inflammatory modulation (Liu et al., 2020). In 2021, the team led by Qiufu Ma further affirmed the scientific validity of acupuncture treatments by shedding light on the neuroanatomy involved in acupuncture's activation of specific signaling pathways (Liu et al., 2021). Moreover, the World Health Organization (WHO) acknowledges acupuncture's efficacy in addressing a wide range of conditions, including but not limited to chronic pain, nausea, and certain neurological disorders. Such recognition further underscores the significance and widespread applicability of acupuncture within contemporary medical practices. In the era of the Internet's evolution, deep learning's pervasive use in daily life has become evident. Its applications span essay recommendations to text data analysis, serving as an essential component (Wang et al., 2018; Guan et al., 2020). The integration of deep learning into the medical domain is equally noteworthy, synergizing neural networks and medical technology to advance science and technology. The surge in regional health informatization and medical technology has amassed substantial medical data. Extracting and effectively utilizing this information is vital for intelligent medical support (Hou et al., 2018). Knowledge graph (KG) technology, as an emerging technology for information organization and processing, demonstrates the ability to efficiently integrate and analyze vast amounts of data and information. By systematically consolidating both ancient and modern literature into a knowledge base, knowledge graphs offer decision-making support for medical practitioners. In the realm of TCM, this technology presents new opportunities for development.

The construction of knowledge graphs is a fundamental concern within the field of knowledge graph research (Liu et al., 2016). The knowledge graph architecture encompasses both its logical structure and technical components. In this paper, we concentrate on two vital aspects of knowledge graph construction: the technical architecture, specifically entity extraction, and relationship extraction.

Nevertheless, constructing high-quality medical knowledge graphs faces significant challenges, particularly due to the distinct characteristics and requirements of medical terminology compared to common terms. Such endeavors often demand substantial human and material resources. In particular, the development of Traditional Chinese Medicine (TCM) knowledge graphs has received relatively less research attention compared to general medical knowledge graphs. Furthermore, detailed research specific to each branch of TCM remains limited.

In the field of acupuncture and tuina, there are still many problems.

- Many acupuncture and tuina treatment plans have many ambiguous concepts, such as the location of acupuncture, the strength of tuina, etc.
- There is a lack of specific quantitative assessment in the implementation of treatment protocols.
- The extant TCM systems are heterogeneous. In the case of acupuncture alone, 26 mainstream schools exist (Zhang and Xia, 2018).

All of the above problems pose a major obstacle to the development of personalized treatment plans. In our previous work, a knowledge graph of acupuncture and tuina was constructed using ancient Chinese medical literature, but there is still no graph filling technique for modern literature (Han et al., 2021).

In the context of Prompt Tuning for Few-shot Named Entity Recognition (NER), the majority of existing methods have primarily focused on English text, resulting in a relative scarcity of methods specifically designed for Chinese text. Chinese sentences tend to be longer compared to English sentences, often spanning tens or even hundreds of Chinese characters. This increased sentence length significantly expands the search space for templates and poses challenges in finding suitable templates for Chinese text. Moreover, Chinese entities typically consist of at least two characters, and in domains like Chinese medicine, entities can even comprise nearly ten words. The diverse meanings of Chinese characters introduce the possibility of unintended partially nested entities within longer entities. All this makes the previous methods do not fit well in Chinese texts.

In order to solve the above problems, in this paper, we hope to accomplish the structured storage and retrieval of acupuncture and tuina knowledge in modern literature with the help of knowledge graphs, and at the same time provide a basis for personalized medical solution recommendation. To summarize the contribution of this work:

- We have collected a large amount of knowledge related to the field of acupuncture and tuina and have built a small domain knowledge base based on this knowledge. It contributes to the construction of the KG later, and also facilitates the needs of other researchers.
- The development of the KG revolves around two primary facets: NER and ERE. For ERE, we opted for the conventional rule-based method for relationship extraction. In contrast, for NER, we devised a hybrid entity extraction model that combines Trie tree-based techniques with deep learning. In particular, we proposed the TemplateFC model, which becomes a more adaptable template-free prompt tuning method for Chinese text by adding BiLSTM layer and CRF layer for joint training.
- We built a knowledge graph of acupuncture and tuina based on modern literature. This graph encompasses 10,346 entities and 440,919 relationships. Additionally, we have developed a user-friendly entity query interface and UI, enhancing accessibility and usability.

**Figure 1:**
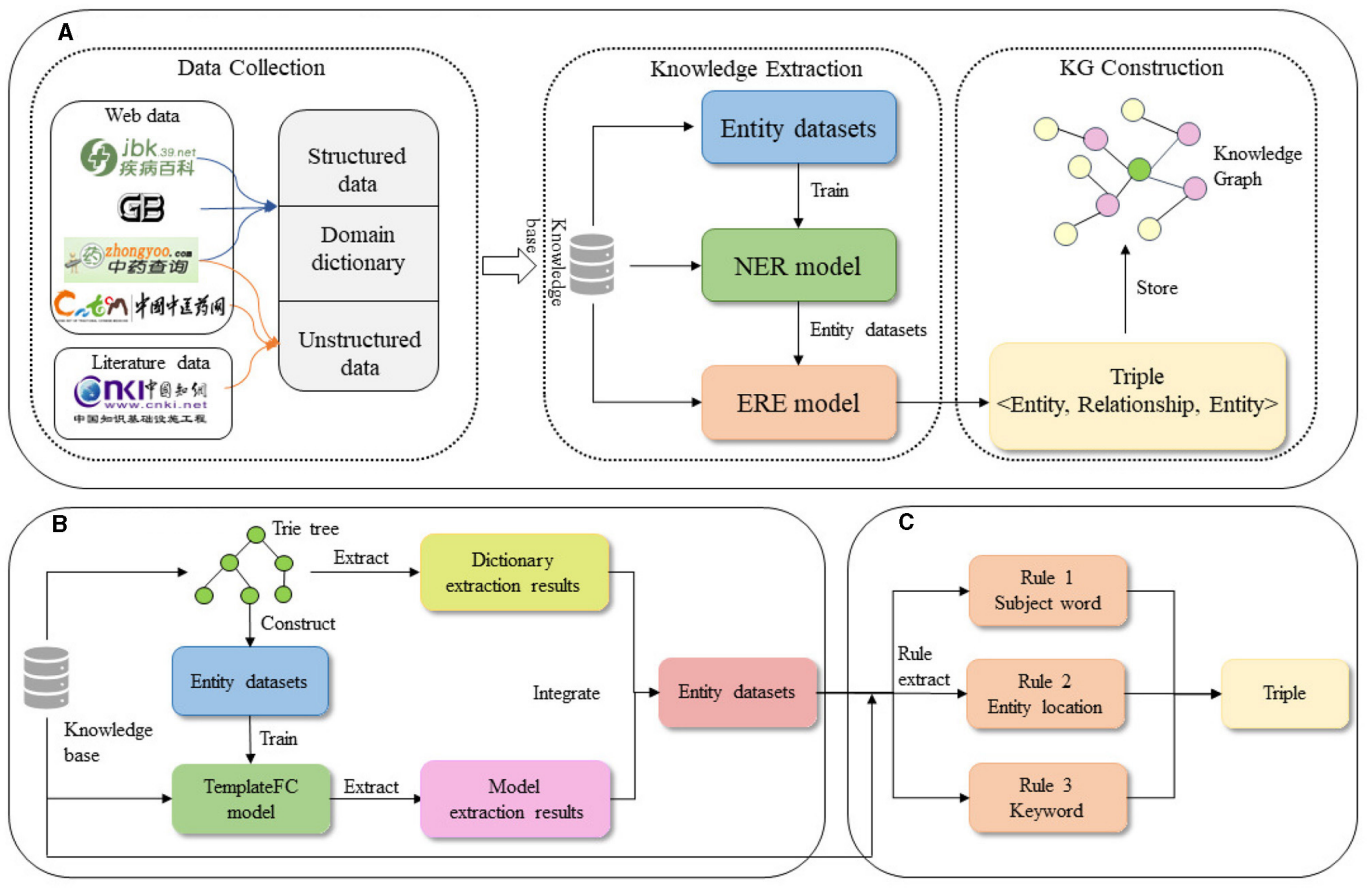
Flow chart of knowledge graph construction. **(A)** The flow of knowledge graph construction. **(B)** Entity extraction. **(C)** Relationship extraction.

The study of this paper is shown in Figure 1.

## 2 Related work

### 2.1 Named Entity Recognition (NER)

Entity extraction, also known as Named Entity Recognition (NER), holds significant importance in the field of natural language processing. Currently, two primary architectural approaches are prevalent in NER research: Neural Network-Conditional Random Field (NN-CRF) and sliding windows. In the NN-CRF architecture, a sequence labeling model, such as CRF, is employed, utilizing a neural network for text representation and feature extraction. Dai et al. (2019) utilized the BERT-BiLSTM-CRF architecture to successfully perform entity extraction for Chinese medical records. Conversely, the sliding window architecture treats NER as a classification task, identifying and classifying potential entities within a sentence. Eberts and Ulges (2019) employed syntactic features to classify Spans created by splitting the sentence. In practical industrial applications, NER techniques often employ hybrid models that combine multiple methods to achieve improved results. Meituan technicians, for instance, employed a combination of lexicon and deep learning models for entity recognition, as documented in the literature. In this paper, we also employ a similar hybrid model.^1^

### 2.2 Prompt tuning for few-shot NER

Prompt learning refers to changing the downstream task to the text generation task by adding some hints to the input of the model without significantly changing the structure and parameters of the pre-trained language model. This approach has gained popularity, particularly in low-resource scenarios of Named Entity Recognition (NER). Numerous authors have proposed their own ideas during this period, leveraging templates tag words, and other techniques. For instance, Lee et al. (2021) focused on selecting specific high-quality examples, while Cui et al. (2021) explored the process of identifying suitable templates. Additionally, Chen et al. (2023) introduced the concept of weighted averaging of multiple prompts using mask-reducible prompts. These advancements have contributed to establishing a strong correlation between the quality of search results and the output of prompt learning techniques.

### 2.3 Entity Relationship Extract (ERE)

Entity Relationship Extraction (ERE) serves as the successor task to entity extraction. Traditional approaches primarily rely on rule-based methods, which identify relationship expressions by detecting specific patterns and using regular expressions. Conversely, deep learning-based methods for relationship extraction closely resemble entity extraction and necessitate a substantial amount of annotated data for supervision. Liu and Zhao (2020) proposed a neural network architecture namely BERT-CNN-BiLSTM-CRE, to achieve relationship extraction in the medical domain. Their architecture was successfully applied to extract relationships from a medical corpus. Moscato et al. (2023) used three biomedical datasets and a multi-task learning framework for relationship extraction. Milošević and Thielemann (2023) have developed a new rule-based method for relationship extraction. The method relies on vocabularies for relationship trigger words, negation cues, speculation cues, mode of action (MoA) cues, and grammar pattern rule set.

### 2.4 Knowledge graph of TCM

The construction of the Traditional Chinese Medicine (TCM) knowledge graph has attracted the involvement of numerous domestic and international internet companies. Yu et al. leveraged the linguistic system of TCM as a foundation and integrated a series of TCM-related databases to develop a comprehensive TCM knowledge graph. This knowledge graph was subsequently embedded and utilized in a TCM knowledge service platform. Furthermore, Yu et al. (2015) capitalized on the digital resources accumulated in the field of TCM to construct a knowledge graph specifically tailored to TCM healthcare. Currently, one of the most notable TCM knowledge graphs is developed by the Institute of Traditional Chinese Medicine Information at the Chinese Academy of Traditional Chinese Medicine. This knowledge graph encompasses 127 semantic types and 58 semantic relationships, establishing it as a valuable resource in the field (Cui et al., 2014). The global acupuncture clinical trial research is booming, and clinical evidence for acupuncture is emerging. Nenggui Xu's team applied artificial intelligence analysis technology to complete the “linking” of original research and 332 systematic evaluations of evidence in 20 disease areas, comprehensively improved the clinical evidence matrix of acupuncture therapy in the Epistemonikos database, and formulated the world's first clinical evidence atlas for acupuncture (Lu et al., 2022).

## 3 Construction of the knowledge graph schema layer

Before the knowledge graph is constructed, it's critical to have a deep understanding of domain requirements. In the field of acupuncture and tuina, the core problem faced is disease and treatment, and the main questions to be addressed are as follows.

- What are the possible symptoms of a disease? What treatment techniques should be used and which acupoints should be targeted?
- What are the possible diseases associated with certain symptoms? What treatment techniques should be used and which acupoints should be targeted to relieve symptoms?
- What are the common therapies of acupuncture and tuina? Which acupoints are commonly used for a particular therapy? What kind of functions will it have?
- What are the common acupoints used for acupuncture and tuina? What are the effects of stimulating a particular acupoint?

After analyzing the aforementioned issues, we have identified five main categories of entities that are crucial to the field of acupuncture and tuina. These categories include disease, symptom, acupoint, therapy, and function. The specific descriptions of each category are summarized in Table 1. In Section 5 of our paper, we specifically address the challenge of dealing with these issues.

**Table 1:** Examples of various types of entities.

**Entity**	**Abbreviation**	**Example**
Disease	DIS	Hypertension, Heart disease
Symptom	SYM	Runny nose, Nasal congestion
Acupoint	XW	Weiling, Sanjiao
Therapy	OPE	Moxibustion, Push
Function	FUN	Reduce fever, Hemostasis

The distinction between diseases and symptoms lacks a clear demarcation. For instance, the entity “headache” can be regarded both as a disease and as a symptom of a disease. To ensure consistency and prevent conflicts during the construction of the knowledge graph, we impose limitations on the scope of diseases. Specifically, all disease entities are required to align with either the International Classification of Diseases, 10th Revision (ICD-10) for [Classification in Health (Australia), 2004] or the Clinical Terminology for Chinese Medicine, Disease Section (GB/T 16751.1-1997). Consequently, if a suspected entity corresponds to a disease or symptom and fulfills both of the mentioned criteria, it is classified as a disease entity; otherwise, it is categorized as a symptom entity.

Both acupoints and meridians play crucial roles in TCM, representing significant theoretical relationships. Meridians are characterized by their linear distribution throughout the body, while acupoints are specific points or zones along these meridians. It can be considered that acupoints are attributed to meridians, including the existence of extra-meridian points. Given the limited number of meridians, for the purpose of this paper, we consider meridians and acupoints as entities of the same type.

Then, this paper also defines six relationships according to the requirements, as described in Table 2.

**Table 2:** Example of each type of entity relationship.

**Category**	**Abbreviation**	**Example**
Associations	DIS-SYM	Symptom
	DIS-OPE	Common therapies for treatment
Treatment	DIS-XW	Commonly used acupoints for treatment
	OPE-XW	Commonly used acupoints for therapy
Function	OPE-FUN	Therapy has the function
	XW-FUN	Stimulation of acupoints has the function

**Figure 2:**
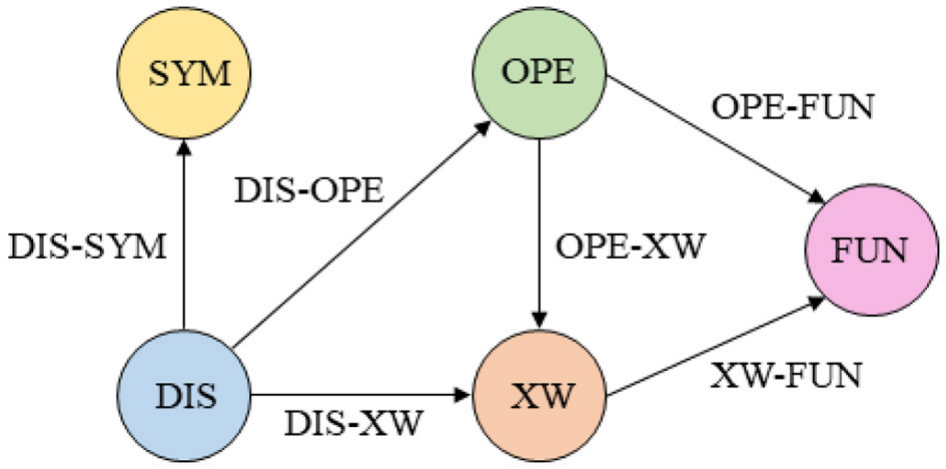
Acupuncture and Tuina knowledge graph entity relationship diagram.

After the above definition, the specific framework design of the acupuncture and tuina knowledge graph schema layer is shown in Figure 2.

## 4 Construction of the domain knowledge base

The cold-start problem poses a significant challenge in the construction of domain knowledge graphs at the current stage. Both entity extraction and relationship extraction rely heavily on annotated data, necessitating the creation of comprehensive datasets. Furthermore, annotators involved in dataset annotation are required to possess substantial domain-specific knowledge.

Hence, prior to constructing the data layer, this paper advocates the establishment of a comprehensive domain knowledge base. This knowledge base encompasses essential components, namely the domain dictionary, disease information base, acupoint information base, and acupuncture and tuina literature base. The integration of these resources aims to address the challenges posed by the limited domain expertise of personnel and mitigates the complexities associated with data annotation processes. We also hope to improve the structure of the graph with the domain knowledge base, including increasing the types of relationships and supplementing entity attributes.

### 4.1 Structured knowledge base

According to the above, we mainly collected data for two types of entities, disease, and acupoint. For the disease entities, information on aliases, onset sites, registered departments, and typical symptoms of common diseases was crawled. A total of 2011 common diseases were crawled, and the sample data (only some attributes of some records were extracted) are shown in Table 3.

**Table 3:** Sample from disease data.

**Disease**	**Section**	**Site of disease**	**Symptom**
Measles	Infectious diseases section; pediatrics	Whole body	Fever
Rhinitis	Otolaryngology	Nose	Runny nose; Stuffy nose; Loss of smell;
Liver cysts	Hepatobiliary surgery	Liver	Abdominal; masses; stomachache

For the acupoint entity, we crawled the data related to acupoint and meridian in the Chinese medicine network.^2^ A total of 366 common acupoints and meridians were crawled, and the sample data (only some attributes of some records were extracted) are shown in Table 4.

**Table 4:** Sample from acupoint data.

**Acupoint**	**Attributed meridians**	**Functions**	**Method of operation**
Atrium	Conception vessels	Unblocking of veins; Tranquility	Flat prick 0.3 0.5 inch
Zuqiaoyin	Gallbladder meridian	Invigorate the meridians: Migraine, Tinnitus	Shallow prick 0.1 inch or prick blood
Yangxi	Large intestine channel of hand yangming	Clearing heat and benefiting the throat; Heartburn; Cataracts of the eyes	Straight prick 0.3 0.5 inch

In order to make the knowledge base scalable and more efficient for retrieval, the obtained data are stored in database in the form of tables to establish a structured knowledge base.

After crawling the structured data, we directly populate it into the knowledge graph as entities or their corresponding relationships. This approach enables us to include some data directly in the initial graph, effectively addressing the cold start problem. As an illustration, consider the second row of data in Table 3, we discern that the symptoms associated with rhinitis include runny nose, stuffy nose, and loss of smell. This information was directly incorporated into the KG, accompanied by the relevant relationships, resulting in the creation of a node graph representing the interconnections among entities linked to rhinitis.

### 4.2 Domain dictionary

Before proceeding with formal entity extraction, it is necessary to collect a representative sample of each entity type in order to build a comprehensive entity dictionary. The entities were primarily sourced from Chinese medicine websites^3^ and other relevant sources. An example dataset for the dictionary is presented in Table 5. To maintain consistency, the length of all entities was limited to 10 characters. It should be noted that not all entities in the knowledge base or domain dictionary were added to the knowledge graph in order to avoid creating a large number of “isolated nodes” and connected components during entity extraction. If the current entity appears in the corpus then it is added to the knowledge graph. Conversely, if it only appeared within the domain dictionary and not in the corpus, it means that it is likely to have no relational triples and is isolated, then it will not be added to the knowledge graph. Finally, only about 48% of the entities were added to the graph.

**Table 5:** Example data for the dictionary.

**Entity**	**Number**	**Example**
Disease	14,691	Enteritis, Pneumoconiosis
Symptom	5,142	Intestinal tinnitus, Dry lips
Acupoint	594	Guanchong, Zhongwan
Therapy	579	Slap, Twist, Push
Function	357	Toning the spleen, stomach,
		liver and kidneys

### 4.3 Unstructured literature base

The corpus data used for information extraction is mainly obtained from three parts: China National Knowledge Infrastructure (CNKI),^4^ Chinese Medicine and Chinese Herbs Network,^5^ and Chinese Medicine Network (see text footnote ^3^). literature for subsequent analysis and knowledge graph construction.

CNKI is a comprehensive repository of knowledge resources in China, covering a wide range of subject areas. In this website, we are able to obtain valuable and authoritative literature related to acupuncture and tuina. However, due to copyright restrictions, we can only download a limited number of relevant documents. To further analyze the literature, we performed optical character recognition (OCR) on the PDF documents, converting them into TXT format to obtain editable text. This allowed us to extract and process the textual content of the literature for subsequent analysis and knowledge graph construction.

Chinese Medicine and Chinese Herbs Network and Chinese Medicine Network are similar open websites for Chinese medicine, with a large number of medical post related to acupuncture and tuina. These posts serve as valuable sources for extracting entities and relationships required for constructing the knowledge graph. To compensate for the limited corpus available on the Knowledge Network, we collected web texts from these medical websites. In total, we crawled 3,236 posts relevant to acupuncture and tuina.

The collected literature underwent basic preprocessing, including the filtering of special symbols. The title, content, and source information of the literature were then stored in the database, establishing a comprehensive library of acupuncture and tuina literature.

## 5 Construction of the graph data layer

### 5.1 Entity extraction based on fusion models

We used the above corpus for entity extraction. It's a combination of the Trie tree model and deep learning model, as depicted in Figure 1B of a diagram. The Trie tree construction relied on the previously built domain dictionary and primarily extracted known entities. The extraction results from the Trie tree served as the dataset for training the deep learning model. The trained model was then used for a second round of entity extraction, focusing on identifying potential entities that were not initially discovered. Finally, the extraction results from both methods were merged to obtain the final entity set.

#### 5.1.1 Entity extraction based on Trie tree

**Figure 3:**
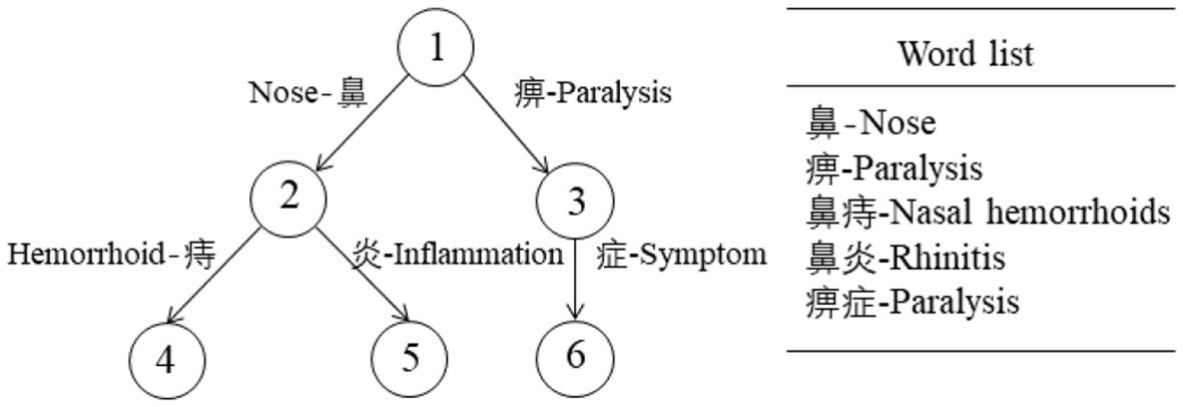
The example of a Trie Tree.

Trie tree, which is also known as dictionary tree and prefix tree. Figure 3 shows a Trie tree and the list of words it contains. For this tree, a query for the entity “rhinitis” would follow the path “1-2-5”.

In this study, the construction of Trie trees was implemented using a Dictionary built in Python. The key-words of the Trie tree are strings, and the Trie tree stores each keyword in a path instead of a node. With different node paths, different keywords were gotten, i.e. entities. In addition, two keywords with a common prefix have the same path in the prefix part of the Trie tree. Here, we modeled a total of five Trie trees, each representing a different entity type, for entity extraction. The algorithm for constructing the Trie trees was described in Algorithm 1.

**Algorithm 1:** AddTrieNode.


**Input:** *root*:*Node, word*:*String*
**Output:** *root*:*Node*
1: *curNode*⇐*root*
2: **for** *c* in *word* **do**
3: **if** *cnotincurNode*.*child* **then**
4: *curNode*.*child*[*c*]⇐*Node*
5: **end if**
6: *curNode*⇐*curNode*.*child*[*c*]
7: *N*⇐*n*
8: *curNode*.*end*⇐*True*
9: **end for**
10: **return** *root*

**Figure 4:**
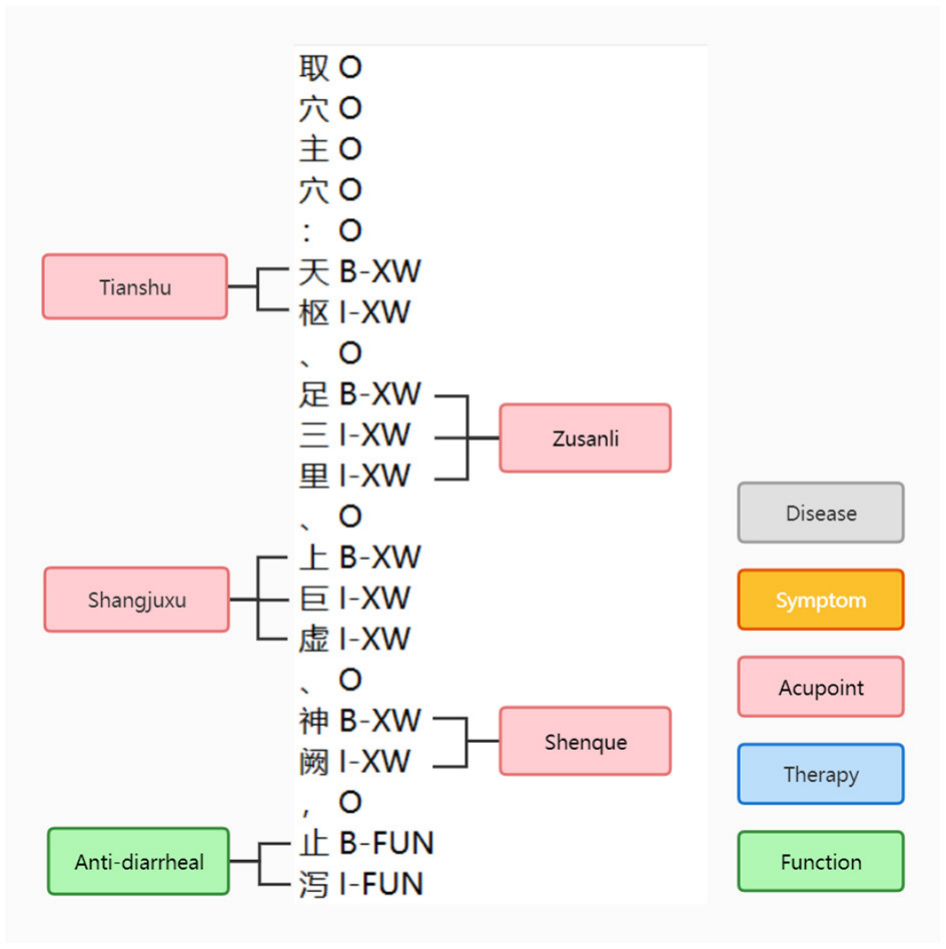
BIO labeling example.

After obtaining the Trie tree, entity extraction and data annotation were performed for the 819 literature samples. The data annotation followed the BIO annotation format. For each literature, it was divided into sentences, and Algorithm 2 was applied to generate candidate words and match them with the Trie tree. The entities that matched successfully were annotated with the corresponding BI tags, while unsuccessful candidates were annotated with the O tag. Entity extraction was conducted for all 819 samples. The number of entity samples obtained for each entity type is presented in Table 6, and the annotation samples can be seen in Figure 4.

**Algorithm 2:** FindEntity.


**Input:** *root*:*Node, sentence*:*String*
**Output:** *entities*:*List*
1: *curNode*⇐*root*; *index*⇐0;*maxLength*⇐10;*entities*⇐[ ]
2: **while** *index*<*len*(*sentence*) **do**
3: *j*⇐*maxLength*
4: **while** *j*≠0 **do**
5: *word*⇐*sentence*[*index*:*index*+*j*]
6: **if** *SearchWord*(*root, word*) = *True* **then**
7: *index*⇐*index*+*j*−1;*entities*.*add*(*word*);
8: **end if**
9: *j*⇐*j*−1
10: **end while**
11: *index*⇐*index*+1
12: **end while**
13: **return** *entities*

**Table 6:** Trie tree extraction entity sample statistics.

**Entity**	**Total**	**DIS**	**SYM**	**XW**	**OPE**	**FUN**
Number	10,346	3,671	3,252	1,491	1,330	602

#### 5.1.2 TemplateFC based entity extraction

##### 5.1.2.1 Dataset pre-processing

The BERT embedding models have a maximum input sequence length of 512 tokens. Considering the two special characters [CLS] and [SEP], the original sequence length should be less than 510 tokens. Since most of the literature in the previous section exceeds this length, we needed to divide the literature.

We opted to divide the literature into blocks while aiming to preserve as much information as possible about the headings at each level. The blocks were created to be as large as possible but still smaller than 510 tokens. Additionally, we retained the requirement of dividing the literature by sentence. As a result, a total of 2505 literature pieces were obtained after the block division. Subsequently, Trie tree-based entity extraction was conducted on the divided literature to obtain the initial training dataset for the deep learning model.

##### 5.1.2.2 TemplateFC model

In the template-free prompt tuning method, NER (Named Entity Recognition) was reimagined as a language modeling (LM) task. However, instead of relying on predefined templates, a new objective called Entity-oriented LM (EntLM) was introduced to fine-tune NER without reusing the LM objective (as done in previous approaches using templates) (Ma et al., 2021). In this method, the LM was trained to predict a label word at the position of the entity when given input text, serving as an indication of the entity's label. For non-entity words like “was” the LM continued to predict the original word.

However, the template-free prompt tuning method also has some disadvantages when applied to the NER task, particularly in the context of Chinese text. Chinese entities often exhibit continuity, leading to situations where a single entity is identified as multiple entities. For instance, in the case of “gastrointestinal neurosis”, the term “gastrointestinal” might be identified as an acupoint entity, while “neurosis” is recognized as a disease entity.This highlights the challenge of accurately identifying and disambiguating Chinese entities due to their structural uniqueness. Another limitation is that the EntLM model is primarily designed for English text and may not be well-suited for Chinese language processing. In English, most entities are typically represented by a single token, whereas Chinese entities often span multiple tokens. This difference in tokenization and entity representation increases the likelihood of inaccurate identification when applying the EntLM model to Chinese text.

In this paper, we proposed an NER model that combines the benefits of template-free prompt learning with enhanced applicability to Chinese text (TemplateFC). We continued to employ a template-free entity-level LM fine-tuning process, but we introduced additional components, namely Bi-directional Long Short-Term Memory (BiLSTM) and Conditional Random Field (CRF) layers, to improve the accuracy of Chinese entity recognition. By incorporating these layers into the training process, the model can learn useful constraints that facilitate better fine-tuning of the pre-trained model.

**Figure 5:**
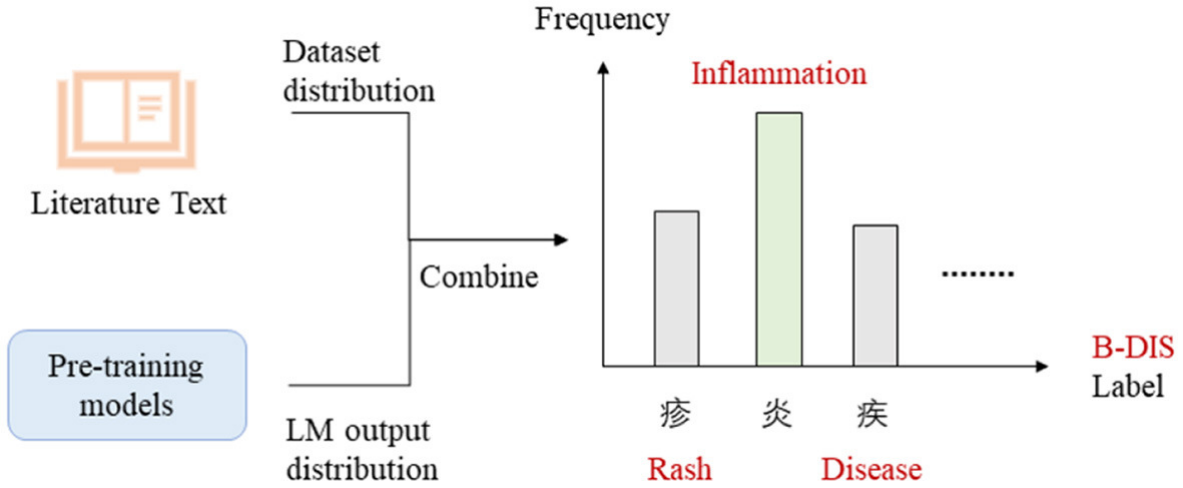
Label words selection.

First, we selected the appropriate label words for the NER model. Due to the limited availability of few-shot data, the label words were chosen randomly. However, to improve the generalizability of the model, we selected label words from a pool of 1,060 documents in the literature base. The selection of label words was performed using the best method from the EntLM model, which combined both Data and LM search. The process of selecting label words for the B-DIS label was illustrated in the Figure 5.

**Figure 6:**
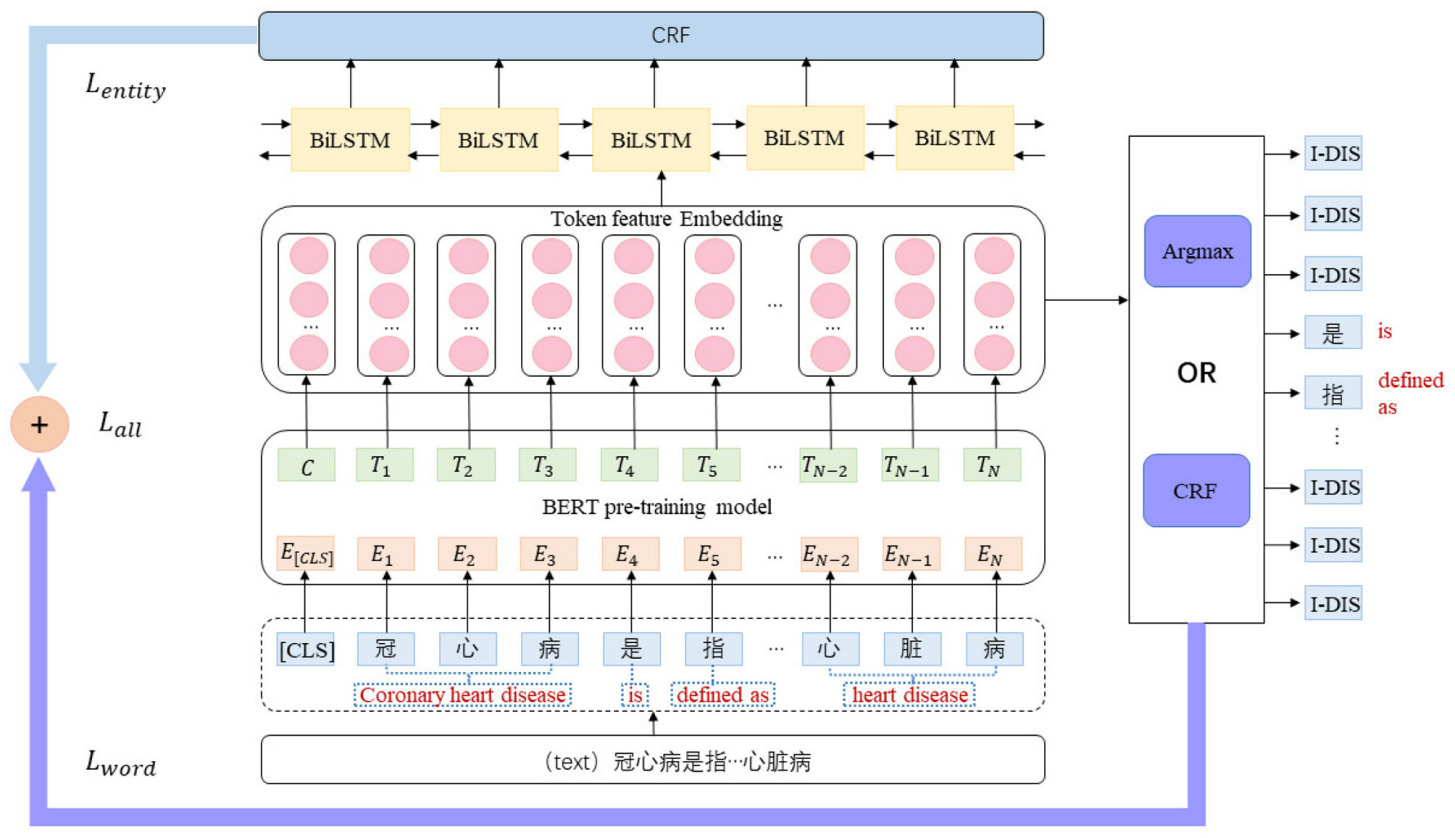
TemplateFC model architecture diagram.

Figure 6 is the general structure of the model. This model first gives the input text *X* = {*x*_1_, …, *x*_*n*_}, and its corresponding label sequence is *Y* = {*y*_1_, …, *y*_*n*_}. Here, we set the set of label words to *V*_*l*_, and it was connected to the set of task labels with a mapping function *M*:*Y*→*V*_*l*_. Next, the target sequence we want to obtain is $X^{Ent}=\left\{x_{1},\ldots ,M\left(y_{i}\right),\ldots ,x_{n}\right\}$ ( Here, we assume that the word at *i* is the entity label). The loss function is shown in Equation 1.


(1) $$\begin{array}{l} L_{word}=-\overset{n}{\underset{i=1}{\sum }}\log P\left(x_{i}=x_{i}^{Ent}|X\right). \end{array}$$

In the following, we set the embedding vector *W* = {*w*_1_, …, *w*_*n*_} obtained after the BERT pre-training model. Next, the embedding vector representation *W* gets the Emission score matrix $X_{r,l}\in R^{n\times l}$ (*n* is the number of tokens, *l* is the number of label types) of the current token location about each label through the BiLSTM layer. Each token may be a different label, so there are *n*×*l* possible paths, and only one true path exists among them. Therefore, after passing through the CRF layer, a new loss function was obtained as shown in Equation 2.


(2) $$L_{entity}=\frac{P_{realpath}}{\sum_{i=1}^{n}P_{i}}.$$

$P_{i}=e^{S_{i}}$ is the score of the *i*-th path. The exact calculation of *S*_*i*_ is shown in Equation 3.


(3) $$\begin{array}{l} S_{n}=EmissionScore+TransitionScore. \end{array}$$

*EmissionScore* can be obtained from the Emission score matrix, and *TransitionScore* is composed of parameters in the CRF layer. Finally, we combined the two loss functions to get the new Loss value and performed joint training, the new Loss value is shown in Equation 4.


(4) $$\begin{array}{l} L_{all}=L_{word}+L_{entity}. \end{array}$$

After the input text *X* got the embedding vector *W* by the pre-training model, the label corresponding to the current token can be obtained directly by the argmax function, as shown in Equation 5.


(5) $$\begin{array}{l} Y=argmax\left(x_{i}=x_{i}^{Ent}|X\right). \end{array}$$

Alternatively, the corresponding label can be obtained by decoding through the CRF layer.

#### 5.1.3 Fusion of entity extraction results

When the entity extraction based on the Trie tree and ALBERT-CRF were completed, the results of the two extractions needed to be fused. Drawing on the literature, and Meituan's design of the entity extraction model, we used dictionary matching to mine discovered entities and deep learning models to mine potential entities. The following fusion rules were developed.

- When the Trie tree extraction results agree with the model extraction results, the results are fused directly.
- When the Trie tree extraction result is “O” and the model extraction result is “B-” or “I-”, the fusion result will be based on the model extraction result.
- When the Trie tree extraction result is “B-” or “I-” and the model extraction result is “O”, the fusion result will be based on the Trie tree extraction result.

The specific fusion algorithm is shown in Algorithm 3.

**Algorithm 3:** Blind.


**Input:** *entity*:*String, label*1:*String, label*2:*String*
**Output:** *entity*:*String, label*:*String*
1: *curEntity*⇐*entity*; *curLabel*1⇐*label*1;*curLabel*2⇐*label*2
2: **if** *curLlabel*1 = *curLabel*2 **then**
3: *label* = *curLlabel*1
4: **else if** *curLabel*1 = \*O*″*and*(*curLabel*2 = \*B*−″*orcurLabel*2 = =\*I*−″) **then**
5: *label* = *curLlabel*2
6: **else if** (*curLabel*1 = \*B*−″*orcurLabel*1 = \*I*−″)*andcurLabel*2 = \*O*″ **then**
7: *label* = *curLlabel*1
8: **else**
9: *break*
10: **end if**
11: **return** *label*

Finally, a total of 10346 entities were extracted from all the literature for the fusion model. Among them, 3671 are DIS entities, 3252 are SYM entites, 149 are XW entities, 1330 are OPE entities, 602 are FUN entities.

### 5.2 Rule-based relationship extraction

Acupuncture and tuina constitute an ancient and specialized medical domain characterized by a stable terminology and conceptual framework. Given the absence of an initial relational dataset and the challenge of acquiring extensive labeled data, opting for a rule-based approach becomes advantageous. This approach involves extracting relational patterns based on expert experience and existing literature, thereby enhancing alignment with the specialized nature of acupuncture and massage. Additionally, it serves to alleviate the burden associated with data labeling. To establish an expression paradigm for the relationships, a substantial amount of text was analyzed and summarized. Subsequently, we devised a set of coherent matching rules to facilitate the extraction of relationships. The formulation of rules primarily encompassed subject word-based extraction, entity location-based extraction, and keyword-based extraction strategies. Our rules were built based on a large amount of text and facts. For example, “Body acupuncture and massage with acupuncture operation: Quchi, Hegu and matching acupoints”, our model identified body acupuncture and massage as OPE entities, identified Quchi and Hegu as XW entities, and extracted the corresponding OPE-XW relationships.

This paper combines three common rule-based approaches and an analysis of literature data to develop rules as shown in Table 7.

**Table 7:** Relationship extraction rules.

**Category**	**Rule**	**The relationship of extraction**
Subject word	Disease therapy literature	DIS-SYM, DIS-OPE, DIS-XW
	Introduction to therapy literature	DIS-OPE, OPE-XW, OPE-FUN
Entity location	Proximity of hand techniques and acupoints	OPE-XW
Keyword	“efficacy”, “effect”, “function”	OPE-FUN, XW-FUN

A total of 40,919 relationships were extracted from all the literature. Among them, 5566 are DIS-SYM relationships, 6705 are DIS-OPE relationships, 6412 are DIS-XW relationships, 6808 are OPE-XW relationships, 6824 are OPE-FUN relationships, 8604 are XW-FUN relationships.

## 6 Experiments and results

### 6.1 Datasets and implementation details

This paper addressed the limitation of resources in the TCM domain by conducting two experiments: one in a resource-rich setting and another in a few-shot setting. To evaluate our approach, we utilized two datasets from distinct domains, the CoNLL2003 dataset (Sang and De Meulder, 2003) sourced from the newswire domain, and a self-built database specific to the acupuncture and tuina domain within TCM. The details of these experiments were provided below.

**Multiple-shot NER Dataset:** The dataset used in this study was primarily derived from the CoNLL2003 dataset. The textual content of this dataset focused on the People's Daily, which is one of the most influential newspaper publications in China. The dataset comprised three common entity types: Person (PER), Place (LOC), and Organization (ORG) with 25862 training sets, 4671 test sets, and 2,385 validation sets.

**Fewshot-NER Dataset:** This dataset is a 10-shot dataset extracted manually from the above Multiple-shot NER Dateset.

**ZJTA Dateset:** This dataset was constructed based on the previous method for NER and ERE. It comprises five entity types and six relationship types, resulting in a total of 10,346 entities and 40,919 relationships. It is important to note that due to limited availability of public information and restricted access to Chinese medicine acupuncture and tuina, the dataset may be limited in size. Consequently, for the few-shot experiment, we selected a subset of this data. Specifically, the few-shot experiment utilized 10 training texts and 498 test texts.

Finally, We selected the F1 score as the evaluation metric for our experiments. The F1 score, which is a balanced measure of precision and recall, effectively reflects the performance of our experiments in a fair and comprehensive manner.

### 6.2 Baselines and proposed models

In our experiments, we have chosen the richer baselines with the following details.

**Bert + HMM:** A classical model which combines BERT and HMM for NER. It learns the representation of the input text using BERT, which converts each word or subword into a high-dimensional vector representation capturing its rich semantic information. And then models the sequence of BERT representations using the HMM model, which can be used for NER.

**TemplateNER** (Cui et al., 2021): A Prompt Learning approach: using generative BART models for sequence annotation tasks and exploring the potential of BART models for few-shot scenarios using a Template-based approach.

**Two-tower** (Ma et al., 2022): The Few-shot Named Entity Recognition (NER) problem was addressed using a two-tower model. The model comprises two BERT encoders: one encoder is responsible for encoding the representation of each token, while the other encoder encodes the natural language form of the BIO label to obtain the label representation. Subsequently, the model predicts the similarity between each token and all the label representations within the text. Finally, the label with the highest similarity is assigned to the token.

**EntLM** (Ma et al., 2021): A approach which abandoned the template and used NER as a language model task. In NER task, the position of the entity was predicted as label word, and the non-entity position was predicted as the original.

**EntLM-CRF:** Add CRF layer decoding on top of the above model.

**TemplateFC:** The proposed method.

Table 8 presents the results of the proposed method and the baseline approaches across different settings. Here, the bold values represent the highest experimental results in the current dataset. Based on the findings from the table, the following observations can be made.

**Table 8:** Comparison of F1 score results for different resources under two data domains.

**Method**	**Multiple-shot**	**Few-shot**	**ZJTA**
Bert+HMM	0.8879	0.1862	0.2025
TemplateNER	0.8942	0.2945	0.3193
Two-tower	0.9102	0.2893	0.3107
EntLM	0.9041	0.3373	0.3871
EntLM-CRF	0.9039	0.3543	0.4016
TemplateFC (Ours)	**0.9174**	**0.3746**	**0.4268**

1. Our model demonstrates higher scores on the common dataset, both in multiple-shot and few-shot conditions. There are several reasons why the TemplateFC model demonstrates superiority. Firstly, the incorporation of BiLSTM and CRF layers guides BERT to learn more nuanced representations by leveraging sequence label information. This aids BERT in comprehending the structural and semantic nuances within input sequences, particularly when data is scarce, thereby providing supplementary supervisory signals. Secondly, the BiLSTM and CRF layers enable the back-propagation of gradients from label prediction errors, facilitating BERT in adapting its representation learning process based on erroneous predictions. This joint training methodology expedites BERT's convergence to enhanced representations with fewer samples. Lastly, the BiLSTM and CRF layers contribute to BERT's improved understanding of context and sequence continuity, resulting in more coherent and semantically enriched representations.
2. In relation to the self-created dataset in this paper, our model exhibits superior performance, surpassing EntLM-CRF by 2.5%. This underscores the advantages of integrating the BiLSTM and CRF layers for joint training, leading to enhanced model fitting speed, improved NER accuracy, and heightened model generalization.
3. Even in scenarios with abundant resources, our model maintains an advantage of 0.72% compared to the highest-scoring baseline model. This highlights the versatility of our approach, which is applicable across both few-shot and multi-shot scenes. Despite the potential risk of overfitting in multi-shot scenarios, our experimental findings indicate that our model continues to enhance model performance and stability.
4. It is important to note that for the last three baselines, we conducted four training runs to obtain four sets of results. Subsequently, we selected the highest F1 score among the results for comparison. Conversely, for the experiments conducted in this paper, we directly decoded the outputs in the multiple-shot scenario. In the few-shot resource scenario, we employed CRF decoding.

### 6.3 Implementation of the knowledge graph

After the completion of entity extraction, the obtained set of entities and relational triples were stored in Neo4j, a graph database. To facilitate querying and exploration, graph queries were executed using the Cipher language. Additionally, to cater to the needs of non-specialists, a Python query interface.and a web-based query user interface (UI) were developed.

Considering the practical applicability in real-life scenarios, queries were primarily conducted for four main types of entities: diseases, symptoms, acupoints, and therapies. The queries were limited to a step size of 1, which corresponds to single-hop queries. This approach enabled efficient retrieval of related information within a single query operation.

After completing the development phase, the website was deployed locally using a personal computer. Subsequently, local testing was conducted to ensure its functionality.

**Figure 7:**
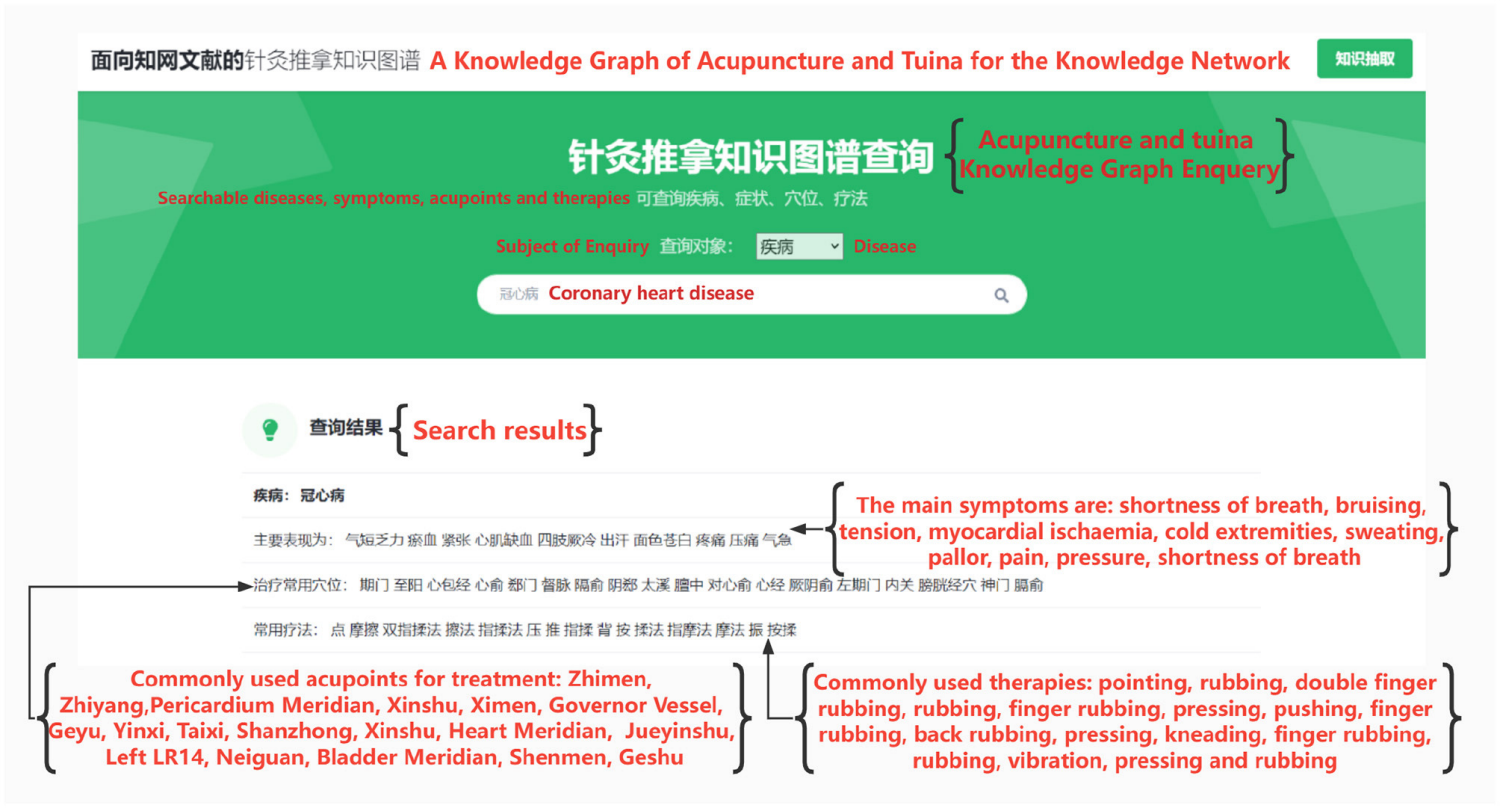
Coronary heart disease text extraction results.

During the entity search, we entered the query “coronary artery disease”, and the results are displayed in Figure 7. The diagram illustrates that coronary artery disease is associated with symptoms such as shortness of breath and sweating. Moreover, commonly employed treatments include pointing, pressing, and kneading. Lastly, specific acupoints such as Zhimen and Zhiyang can be targeted for treatment. This effectively addresses question one as outlined in section two.

To substantiate the credibility of our research, we have opted to engage a reputable medical platform, Hua Tuo GPT. We have meticulously designed a set of three targeted inquiries to be posed and elucidated on this platform:

- What are the main symptoms of coronary heart disease?

**Answer:** The principal manifestations encompass *angina* or *discomfort in the chest*, sometimes accompanied by secondary symptoms like *dyspnea* and *diaphoresis*. In severe instances, symptoms can escalate to *myocardial infarction*. Urgent medical attention is imperative upon experiencing any of these signs, ensuring prompt mitigation of health risks. Furthermore, proactive lifestyle adjustments, such as smoking cessation, alcohol moderation, weight maintenance, and increased physical activity, can collectively serve as preventative measures against disease onset and progression.

- What are the common therapies for the treatment of coronary heart disease?

**Answer:** The treatment paradigm encompasses both pharmacological and non-pharmacological interventions. Pharmacotherapy predominantly targets pain alleviation and cardiovascular function regulation through agents like *anticoagulants, lipid-lowering agents*, and *vasodilators*. Conversely, non-pharmacological avenues involve dietary restructuring, appropriate physical exertion, psychological counseling, among others, which collectively alleviate symptoms and foster recuperation. Importantly, tailored application of a multitude of treatment approaches yields optimal outcomes across diverse coronary heart disease presentations and severities.

- What acupoints to be targeted and what techniques to be used to relieve coronary heart disease?

**Answer:** Acupoints include the *Xinshu* and *Danzhong*. Commonly used acupuncture techniques include *tonifying deficiency, clearing the heart, calming asthma*, and *warming yang*, while tuina focuses on regulating qi and blood, promoting blood circulation for removing obstruction in collaterals, and other aspects. However, due to the variability of each individual's body and different disease states, the choice of acupoints and techniques may vary. Therefore, it is recommended that you consult with a professional practitioner and obtain the necessary assessment and guidance before undergoing acupuncture and tuina treatment.

After analyzing the three provided questions and their corresponding answers, several conclusions can be deduced:

- Enhanced Detail and Precision: Our responses exhibit a notable augmentation in both detail and specificity. For instance, in elucidating the symptoms of coronary heart disease, our answers encompass a broader spectrum of symptoms, encompassing pallor and bruising, which the website's responses did not encompass. This augmentation in symptom coverage enhances the comprehensiveness of our answers.
- Domain-Specific Focus: Our answers distinctly exhibit a focused domain expertise, centered around the realm of acupuncture and tuina. This is reflected in the knowledge graph that this paper presents, which is intrinsically tailored to the acupuncture and tuina domain. Conversely, the website's responses tend to encompass a more generalized medical perspective, devoid of the intricate nuances specific to acupuncture and tuina. This divergence underscores our paper's potency in providing in-depth insights within the acupuncture and tuina domain.

In summation, our website notably excels in the sphere of acupuncture and tuina, securing a distinct advantage over the comprehensive medical advice provided by the website. Our work leverages its domain-specific focus to furnish detailed and precise counsel, aligning with the depth and expertise inherent in the acupuncture and tuina field. This renders our website a preeminent source of tailored advice within the realm of acupuncture and tuina, underscoring its authority and value within this specialized domain.

## 7 Discussion

This paper presents the establishment of a novel knowledge base in the domain of acupuncture and tuina, utilizing modern literature as the foundation. Subsequently, the schema layer of the acupuncture and tuina knowledge graph was designed, considering the requirements for practical applications in TCM. The NER task was accomplished through fused Trie extraction and model extraction techniques, while the ERE task was completed using rule-based methods. Additionally, in the context of few-shot learning, we proposed a TemplateFC model, which becomes a more adaptable template-free prompt tuning method for Chinese text by adding BiLSTM layer and CRF layer for joint training. Lastly, the work encompassed graph storage and querying, enabling the KG of acupuncture and tuina to facilitate doctors' understanding of relevant knowledge and give diagnostic and therapeutic advice.

The method proposed in this paper aims to organize and integrate various knowledge and concepts within the acupuncture and tuina domain, culminating in a structured knowledge graph. This facilitates knowledge sharing and communication among experts and researchers from diverse fields, fostering cross-disciplinary collaboration and discourse. Nonetheless, certain limitations persist in this study. For instance, within ERE, the conventional rule-based approach struggles to encompass all text features adequately, posing challenges for migration. In future work, we intend to explore deep learning-based methods for relationship extraction, tailored to the nuances of Chinese text, thereby advancing TCM development. Concurrently, we aim to delve into the capabilities of Large Language Models (LLMs), aspiring to enhance the precision in the extraction of entities and their interrelationships within textual datasets. The ultimate objective of this exploration is to attain zero-shot learning capabilities, thereby significantly advancing the efficacy and adaptability of our models in understanding and processing complex textual information.

## Data availability statement

The raw data supporting the conclusions of this article will be made available by the authors, without undue reservation.

## Author contributions

XL: Conceptualization, Funding acquisition, Methodology, Project administration, Writing — original draft, Writing — review & editing, Data curation, Formal analysis, Investigation, Software, Validation, Visualization. XH: Conceptualization, Funding acquisition, Methodology, Project administration, Writing — original draft, Writing — review & editing, Resources, Supervision. SW: Data curation, Software, Validation, Writing — original draft, Writing — review & editing. YL: Project administration, Supervision, Writing — original draft, Writing — review & editing. RG: Conceptualization, Funding acquisition, Writing — original draft, Writing — review & editing.

## Footnotes

^1^Exploration and Practice of NER Technology in Meituan Search. https://tech.meituan.com/2020/07/23/ner-in-meituan-nlp.html. Acessed:2024-01-10.

^2^Acupuncture and Tuina - Acupuncture techniques, acupuncture treatment, meridians and acupoints, TCM Tuina. http://www.pharmnet.com.cn/tcm/zjdq/. Acessed:2022-05-09.

^3^Complete List of Human Acupuncture Points—Chinese Medicine Search. http://www.zhongyoo.com/xw/. Acessed:2022-05-04.

^4^Search—China Knowledge Network. https://kns.cnki.net/kns/brief/default_result.aspx. Acessed:2022-05-09.

^5^TCM.com_TCM.com_Excellent Chinese Medicine Portal. https://www.zhzyw.com/. Acessed: 2022-05-09.
